# Levels of adhesion molecules and clinical outcomes in patients with ischemic stroke after mechanical thrombectomy

**DOI:** 10.3389/fneur.2022.1024162

**Published:** 2022-09-29

**Authors:** Xiaohao Zhang, Feng Zhou, Wei Wang, Yan E, Shuaiyu Chen, Haiming Cao, Huiwen Lian, Teng Jiang, Yingdong Zhang, Hongchao Shi, Junshan Zhou

**Affiliations:** ^1^Department of Neurology, Nanjing First Hospital, Nanjing Medical University, Nanjing, China; ^2^Department of Neurology, Jinling Hospital, Medical School of Nanjing University, Nanjing, China

**Keywords:** adhesion molecules, ischemic stroke, thrombectomy, mediation analysis, vascular cell adhesion molecule-1

## Abstract

**Background and purpose:**

Data on adhesion molecule levels in patients treated with mechanical thrombectomy (MT) are scarce. We aimed to evaluate the association among adhesion molecule levels, symptomatic intracranial hemorrhage (sICH), and clinical outcome and to determine whether the sICH influences the association of adhesion molecules with functional outcome.

**Methods:**

Patients with large artery occlusion in the anterior circulation and treated with MT were prospectively recruited. Adhesion molecules, such as soluble intercellular adhesion molecule-1, soluble vascular cell adhesion molecule-1 (sVCAM-1), and soluble E-selectin (sE-selectin) were tested. An unfavorable outcome was defined as a 90-day modified Rankin Scale (mRS) score of 3–6. The sICH was diagnosed according to the Heidelberg Bleeding Classification within 72 h of endovascular treatment (EVT).

**Results:**

Of the 310 enrolled patients (mean age, 68.5 years; 198 men), 46 (14.8%) experienced sICH and 173 (55.8%) experienced an unfavorable outcome at 90 days. After adjusting for potential confounders, patients with higher sVCAM-1 and sE-selectin levels had an increasing trend of sICH [4th quartile vs. 1st quartile for sVCAM-1; odds ratio (*OR*), 2.766, *p* = 0.085; sE-selectin; *OR*, 2.422, *p* = 0.086] and poor outcome (4th quartile vs. 1st quartile for sVCAM-1; *OR*, 2.614, *p* = 0.025; sE-selectin; *OR*, 2.325, *p* = 0.046). Furthermore, the sICH might partially mediate the worse functional outcome in patients with higher adhesion molecules levels (Sobel test, *p* < 0.001 for sVCAM-1 and *p* = 0.007 for sE-selectin).

**Conclusions:**

There were significant relationships between levels of adhesion molecules and a 90-day poor outcome in patients with ischemic stroke treated with MT, which was partially mediated by sICH.

## Introduction

Multiple meta-analyses and randomized studies have supported mechanical thrombectomy (MT) as the standard of care for acute large vessel occlusive stroke in anterior circulation (1, 2). One of the main reasons for the success of MT in treating ischemic stroke is the high rate of revascularization (3). However, despite the successful revascularization of large vessel occlusive stroke, nearly half of patients might experience an unfavorable functional outcome (1, 4–7). Therefore, determining the predictive factors for clinical outcomes is of vital importance for illustrating the mechanisms of ischemia-reperfusion injury and continuously improving the prognosis of patients with ischemic stroke after MT.

The overexpression of adhesion molecules plays an important role in the model of cerebral ischemia-reperfusion injury, including the activation of endothelial cells, disruption of the blood-brain barrier, and transendothelial migration of leukocytes into the brain tissues (8–11). Several studies have evaluated the prognostic value of adhesion molecule concentrations in patients with ischemic stroke, but the results were inconsistent (12–15). Recently, data from the BACTRAC program demonstrated that the intracranial vascular cell adhesion molecule-1 (VCAM1) was positively related to infarct volume, edema volume, and stroke severity in patients receiving MT treatment (16). Symptomatic intracranial hemorrhage (sICH) is a feared procedural complication of MT, which was reported as high as 13.8% in the real-world study (4, 17). Interestingly, it has also been postulated that increasing adhesion molecules may induce endothelial dysfunction and further exacerbate the hemorrhage transformation after stroke (18). Therefore, it is possible that sICH may facilitate the presence of worse functional outcomes *via* altered adhesion molecule levels. However, to date, no study has been conducted to examine the associations of circulating adhesion molecules concentrations with sICH and 90-day functional outcomes in patients with large vessel occlusive stroke after MT.

This prospective study aimed to evaluate (1) the cross-sectional association of adhesion molecules with sICH and a 90-day poor outcome among patients treated by MT; and (2) the mediating effect of sICH on the association between adhesion molecules and functional outcomes.

## Materials and methods

### Study population

During September 2019 and July 2021, patients with acute ischemic stroke undergoing MT for large vessel occlusion were prospectively recruited from Jinling Hospital and Nanjing First Hospital. Patients were included if they: (1) were aged ≥18 years; (2) had a pre-stroke modified Rankin scale (mRS) score < 2; (3) had large artery occlusion in the internal carotid artery or M1/M2 segment of the middle cerebral artery; and (4) were treated with a stent-like retriever and/or an aspiration system. The exclusion criteria for this study were the following: (1) patients who had received intra-arterial thrombolysis alone; and (2) patients who were diagnosed with a concomitant aneurysm, arteriovenous malformation, moyamoya disease, or hematological system diseases. The study was approved by the ethics committee of Jinling Hospital and Nanjing First Hospital, and written informed consents were obtained from all patients.

### Clinical data collection

The following baseline clinical variables, angiographic characteristics, and procedural data were included in this analysis: age, gender, traditional vascular risk factors, blood pressure, baseline blood glucose on admission, baseline stroke severity, pre-treatment infarct volume, cause of stroke, occlusion site, collateral status, procedural modes, and passes of stent retriever device. The baseline neurological deficits were evaluated by the National Institutes of Health Stroke Scale [NIHSS] (19). The Alberta Stroke Program Early Computerized Tomography Score [ASPECTS] was used to detect the infarct volume before MT (20). Stroke etiology was classified according to the criteria of the Trial of Org 10172 in Acute Stroke Treatment (TOAST) (21). The collateral circulation extent was assessed by the American Society of Interventional and Therapeutic Neuroradiology/Society of Interventional Radiology grading system (22), and divided into poor (grades 0–1) or good (grades 2–4). The successful reperfusion was defined as a modified Thrombolysis in Cerebral Infarction score of 2b−3 (4, 6).

### Measurement of adhesion molecules

Venous blood was drawn within 24 h from admission. Serum biomarkers of adhesion molecules, such as soluble intercellular adhesion molecule-1 [sICAM-1], soluble vascular cell adhesion molecule-1 [sVCAM-1], and soluble E-selectin [sE-selectin] were assessed using commercially sandwich immunoassay kits (23). In this study, intra- and inter-assay coefficients of variation were, respectively, 6.0 and 7.0% for sICAM-1, 1.4 and 5.2% for sVCAM-1, and < 10.0 and < 10.0% for sE-selectin.

### Assessment of outcomes

A computed tomography (CT) scan was performed at 24–72 h after endovascular treatment (EVT) or whenever the clinical symptom deteriorations. According to the Heidelberg Bleeding Classification (24), sICH was diagnosed if the new observed intracranial hemorrhage was correlated to any of the following conditions: (1) the NIHSS score increased by more than 4 points compared with the score immediately before worsening; (2) the NIHSS score increased by more than 2 points in 1 category; and (3) deterioration led to intubation, hemicraniectomy, external ventricular drain placement, or any other major interventions. Furthermore, symptom deteriorations could not be explained by causes other than the observed intracranial hemorrhage. Patients were followed up 90 days after stroke onset at the outpatient stroke clinic or *via* telephone by neurologists who were blind to the clinical data. Poor outcome was defined as an mRS score of 3–6 (5, 6).

### Statistical analysis

Analyses were conducted using the statistical software SPSS version 24.0 (IBM, New York, NY) and R version 4.0.0. Continuous variables are presented as means ± SD (normal distribution) and as median with interquartile range [IQR] (skewed distribution). Categorical variables are presented as percentages. We used the chi-square test (or the Fisher exact test when appropriate) and the *t*-test (or the Mann–Whitney *U*-test when appropriate) for categorical and quantitative variables comparisons, respectively. Multiple imputations with chain equations were performed to account for missing values. A binary logistic regression analysis was conducted to evaluate the relationships between adhesion molecules and outcomes after adjusting for demographic characteristics and variables with a *p*-value < 0.1 in the univariate analysis. A sensitivity analysis was also performed to test the robustness of our findings after excluding patients who were lost to follow-up at 90 days. The calculation of odds ratio (*OR*) with 95% confidence intervals (*CI*s) was assessed in all tests. We further explored the pattern and magnitude of the association of adhesion molecules with poor outcomes using the restricted cubic splines with three knots (at 5th, 50th, and 95th percentiles) (25, 26).

In addition, we utilized the mediation analysis to determine whether the sICH influences the association between adhesion molecules and the mRS score (27). The Sobel test was used to determine the statistical significance of the mediation effect (28). All statistical tests were two-sided, and a *p-*value < 0.05 was considered statistically significant.

## Results

A total of 310 patients were included in the study. The mean age was 68.5 ± 12.4 years, and 63.9% (198/310) of patients were men. The median baseline NIHSS score was 13.0 and the median of ASPECTS was 9.0. The median time from a puncture to recanalization was 360.0 min. Using the TOAST criteria and post-procedural images, 47.7% (148/310) of patients with large artery occlusion were diagnosed with the atherosclerotic disease. In total, 95 patients (30.6%) received a combination with rescue therapy.

Using the last observation carried forward, 14 (4.5%) patients' mRS at discharge was recorded as 90 days follow-up. The proportion of patients with mRS of 3–6 at 90 days was 55.8% (173/310). Table 1 presents the baseline characteristics of these patients stratified by the clinical outcome. On univariate analysis, patients with poor outcomes were older than patients without it (71.2 ± 11.9 vs. 65.2 ± 12.4 years; *p* < 0.0001). Poor collateral status (56.6 vs. 41.6%; *p* = 0.009) and sICH (25.7 vs. 1.5%; *p* < 0.001) were more common in patients with a 90-day unfavorable outcome than in patients without it. The successful reperfusion ratio in patients with poor outcomes was lower than in patients without poor outcomes (80.3 vs. 94.9%; *p* < 0.001). Patients with poor outcomes had lower baseline ASPECT scores (median, 8.0 vs 9.0 score; *p* < 0.001) and higher baseline NIHSS scores (median, 15.0 vs. 11.0 score; *p* < 0.001). Baseline blood glucose levels (8.2 ± 3.3 vs. 6.6 ± 2.8 mmol/L; *p* < 0.001) and passes with retriever (median 2.0 vs. 1.0; *p* < 0.001) were higher in patients with poor outcomes than in patients without it. As for adhesion molecules, there were significantly higher values of sVCAM-1 (median 6.4 vs. 5.6 pg/ml; *p* = 0.002) and sE-selectin (median 8.0 vs. 6.3 pg/ml; *p* < 0.001) in the poor outcome group. However, the difference in sICAM-1 (median 1.6 vs. 1.4 pg/ml; *p* = 0.439) between patients with and without poor outcomes did not achieve statistical significance.

**Table 1:** Comparison of baseline data according to patients with and without a 90-day poor outcome.

**Variables**	**All patients, *n* = 310**	**Good outcome, *n* = 137**	**Poor outcome, *n* = 173**	***p-*Value**
**Demographic characteristics**
Age, years	68.5 ± 12.4	65.2 ± 12.4	71.2 ± 11.9	< 0.001
Male, *n* (%)	198 (63.9)	93 (67.9)	105 (60.7)	0.191
**Vascular risk factors**, ***n*** **(%)**
Hypertension	216 (69.7)	97 (70.8)	119 (68.8)	0.701
Diabetes mellitus	72 (23.2)	27 (19.7)	45 (26.0)	0.192
Hyperlipidemia	30 (9.7)	13 (9.5)	17 (9.8)	0.920
Atrial fibrillation	140 (45.2)	57 (41.6)	83 (48.0)	0.263
Current smoker	119 (38.4)	52 (38.0)	67 (38.7)	0.890
Coronary heart disease	44 (14.2)	19 (13.9)	25 (14.5)	0.884
**Clinical data**
Systolic blood pressure, mmHg	136.9 ± 23.4	136.4 ± 23.4	137.4 ± 23.5	0.773
Diastolic blood pressure, mmHg	82.0 ± 14.2	82.8 ± 15.4	81.4 ± 13.3	0.370
Time from puncture to recanalization, min	360.0 (254.0, 555.0)	355.0 (240.5, 553.0)	371.5 (274.0, 558.5)	0.225
Baseline NIHSS, score	13.0 (10.0, 17.0)	11.0 (8.0, 14.0)	15.0 (12.0, 19.0)	< 0.001
Baseline ASPECTS, score	9.0 (8.0, 9.0)	9.0 (8.0, 10.0)	8.0 (8.0, 9.0)	< 0.001
**Stroke etiology**, ***n*** **(%)**
Atherosclerotic	148 (47.7)	70 (51.1)	78 (45.1)	0.408
Cardioembolic	133 (42.9)	53 (38.7)	80 (46.2)	
Others	29 (9.4)	14 (10.2)	15 (8.7)	
Prior intravenous thrombolysis, *n* (%)	136 (43.9)	62 (45.3)	74 (42.8)	0.662
Poor collateral status, *n* (%)	155 (50.0)	57 (41.6)	98 (56.6)	0.009
Successful reperfusion, *n* (%)	269 (86.8)	130 (94.9)	139 (80.3)	< 0.001
sICH, *n* (%)	46 (14.8)	2 (1.5)	44 (25.7)	< 0.001
**Procedural models**, ***n*** **(%)**
Pure thrombectomy	215 (69.4)	92 (67.2)	123 (71.1)	0.454
Need for rescue therapy^*^	95 (30.6)	45 (32.8)	50 (28.9)	
Passes with retriever	1.0 (1.0, 2.0)	1.0 (1.0, 2.0)	2.0 (1.0, 3.0)	< 0.001
**Vascular occlusion site**, ***n*** **(%)**
Internal carotid artery	106 (34.2)	46 (33.6)	60 (34.7)	0.839
Middle cerebral artery	204 (65.8)	91 (66.4)	113 (65.3)	
**Laboratory data**
Baseline blood glucose, mmol/L	7.5 ± 3.1	6.6 ± 2.8	8.2 ± 3.3	< 0.001
Hs-CRP, mg/L	10.4 (3.6, 23.4)	10.4 (4.3, 22.0)	10.3 (3.3, 26.5)	0.854
sICAM-1, pg/ml	1.6 (0.8, 2.2)	1.4 (0.7, 2.3)	1.6 (0.8, 2.2)	0.439
sVCAM-1, pg/ml	6.0 (4.5, 8.0)	5.6 (4.3, 7.4)	6.4 (5.1, 9.1)	0.002
sE-Selectin, pg/ml	7.6 (4.6, 9.7)	6.3 (3.4, 8.3)	8.0 (5.2, 10.4)	< 0.001

ASPECTS, the Alberta Stroke Program Early Computed Tomography Score; Hs-CRP, hyper-sensitive C-reactive protein; NIHSS, National Institute of Health Stroke Scale; sE-selectin, soluble E-selectin; sICAM-1, soluble intercellular adhesion molecule-1; sICH, symptomatic intracranial hemorrhage; sVCAM-1, soluble vascular cell adhesion molecule-1.

* Rescue therapy includes balloon angioplasty, permanent implantation of a stent, intraarterial thrombolysis, or intraarterial tirofiban infusion.

**Figure 1:**
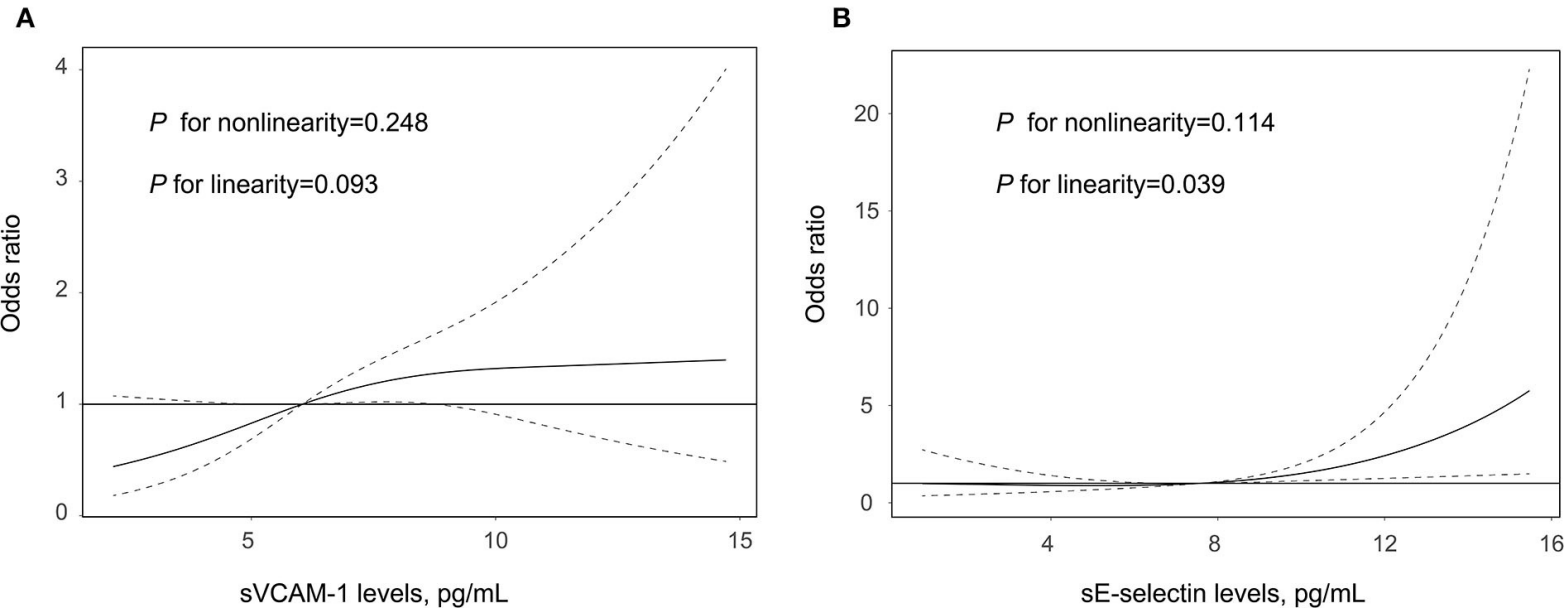
Association of sVCAM-1 **(A)** and sE-selectin **(B)** with the risk of poor outcome after acute ischemic stroke treated by endovascular treatment. Association was fitted with restricted cubic spline with 3 knots (at 5th, 50th, and 95th percentiles) adjusting for covariates with a *p-*value < 0.1 in the univariate analysis. The midpoint of sVCAM-1 and sE-selectin levels were set as the reference point. The solid line represents the odds ratio (*OR*) and the dashed lines represent the 95% confidence interval (*CI*). sE-selectin, soluble E-selectin; and sVCAM-1, soluble vascular cell adhesion molecule-1.

Table 2 summarizes the results of the multivariate regression analysis and the sensitivity analysis for the association between adhesion molecules and clinical outcome. The multivariable logistic regression model suggested that increasing levels of sVCAM-1 (per 1-SD increase; *OR*, 1.308; 95% *CI*: 1.113–1.739, *p* = 0.045) and sE-selectin (per 1-SD increase; *OR*, 1.509; 95% *CI*: 1.079–2.110, *p* = 0.016) were significantly associated with a higher risk of 90-day poor outcome. Similar results were found when the adhesion molecules were analyzed as a categorical variable. In addition, the sensitivity analysis confirmed the robustness of relationships between adhesion molecules and clinical outcomes after excluding the patients without a 90-day mRS. As illustrated in Figure 1, the multiple-adjusted spline regression model further confirmed the dose-response associations of sVCAM-1 (*p* = 0.093 for linearity) and sE-selectin (*p* = 0.039 for linearity) with a 90-day unfavorable outcome.

**Table 2:** The multivariate regression analysis for the associations of adhesion molecule markers with a 90-day poor outcome.

	**Crude model**		**Adjusted model**	
	**OR (95% CI)**	***p-*Value**	**OR (95% CI)**	***p-*Value**
**Logistic regression analysis**
sICAM-1, Per 1-SD increase	1.020 (0.853–1.343)	0.558	1.182 (0.889–1.571)	0.250
sICAM-1 (quartiles)
First quartile	Ref		Ref	
Second quartile	0.833 (0.443–1.566)	0.574	0.975 (0.431–2.208)	0.952
Third quartile	1.543 (0.807–2.951)	0.190	1.641 (0.707–3.808)	0.249
Fourth quartile	0.972 (0.571–1.829)	0.930	1.258 (0.547–2.894)	0.590
sVCAM-1, Per 1-SD increase	1.412 (1.119–1.782)	0.004	1.308 (1.113–1.739)	0.045
sVCAM-1 (quartiles)
First quartile	Ref		Ref	
Second quartile	1.315 (0.714–2.535)	0.359	1.698 (0.750–3.844)	0.204
Third quartile	2.056 (1.074–3.935)	0.029	2.480 (1.069–5.765)	0.034
Fourth quartile	2.782 (1.436–5.392)	0.002	2.614 (1.130–6.048)	0.025
sE-Selectin, Per 1-SD increase	1.744 (1.348–2.056)	< 0.001	1.509 (1.079–2.110)	0.016
sE-Selectin (quartiles)
First quartile	Ref		Ref	
Second quartile	1.328 (0.706–2.500)	0.379	0.710 (0.327–1.545)	0.388
Third quartile	1.725 (0.914–3.258)	0.093	0.860 (0.391–1.890)	0.707
Fourth quartile	3.294 (1.692–6.412)	< 0.001	2.325 (1.040–5.200)	0.040
**Sensitivity analysis**
sICAM-1, Per 1-SD increase	1.104 (0.875–1.392)	0.404	1.215 (0.940–1.631)	0.197
sICAM-1 (quartiles)
First quartile	Ref		Ref	
Second quartile	0.919 (0.481–1.755)	0.798	1.126 (0.472–2.636)	0.802
Third quartile	1.624 (0.840–3.140)	0.150	1.605 (0.669–3.851)	0.289
Fourth quartile	1.085 (0.564–2.080)	0.808	1.386 (0.574–3.344)	0.468
sVCAM-1, Per 1-SD increase	1.479 (1.156–1.891)	0.002	1.385 (1.025–1.871)	0.035
sVCAM-1 (quartiles)
First quartile	Ref		Ref	
Second quartile	1.367 (0.713–2.621)	0.347	1.748 (0.737–4.143)	0.205
Third quartile	2.096 (1.085–4.047)	0.028	2.541 (1.054–6.126)	0.038
Fourth quartile	3.168 (1.594–6.296)	0.001	3.010 (1.242–7.294)	0.015
sE-Selectin, Per 1-SD increase	1.739 (1.334–2.266)	< 0.001	1.433 (1.001–2.051)	0.049
sE-Selectin (quartiles)
First quartile	Ref		Ref	
Second quartile	1.316 (0.689–2.513)	0.406	0.688 (0.307–1.542)	0.364
Third quartile	1.670 (0.874–3.191)	0.121	0.753 (0.322–1.667)	0.459
Fourth quartile	3.331 (1.667–6.659)	0.001	2.389 (1.017–5.608)	0.046

CI, confidence interval; OR, odd ratio; sE-selectin, soluble E-selectin; sICAM-1, soluble intercellular adhesion molecule-1; sVCAM-1, soluble vascular cell adhesion molecule-1.

The adjusting model was controlled for demographic characteristics and variables with a p-value < 0.1 in the univariate analysis including the baseline NIHSS score, pre-treatment ASPECTS, poor collateral status, successful reperfusion, sICH, passes with retriever, and baseline blood glucose.

The sICH was diagnosed in 46 patients (14.8%) within 72 h after MT according to the Heidelberg Bleeding Classification. The results of the comparison of patients with and without sICH are shown in Supplementary Table S1. Patients with sICH had a higher trend of sVCAM-1 (median, 8.1 vs. 7.4 pg/ml, *p* = 0.073) and sE-selectin levels (median, 6.8 vs. 5.9 pg/ml, *p* = 0.025). In the multivariate analysis after adjusting for potential confounders, patients with higher levels of sVCAM-1 (4th quartile vs. 1st quartile; *OR*, 2.766; 95% *CI*: 0.869–8.803, *p* = 0.085) and sE-selectin (4th quartile vs. 1st quartile; *OR*, 2.422; 95% *CI*: 0.822–6.648, *p* = 0.086) had an increasing trend of sICH (Supplementary Table S2).

**Figure 2:**
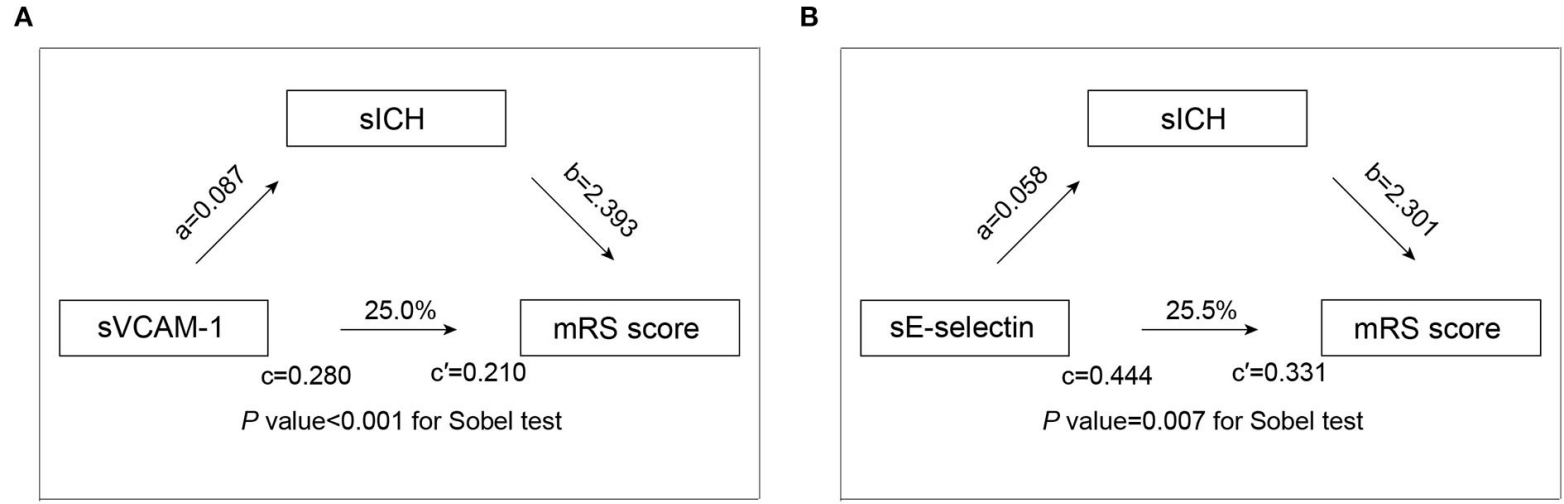
Mediation analysis by sICH on the association of sVCAM-1 **(A)** and sE-selectin **(B)** with functional outcome. a is the regression coefficient of the correlation between adhesion molecules levels and sICH; b is the regression coefficient of the correlation between the sICH and mRS score, using adhesion molecules levels and sICH as independent variables; c is the regression coefficient of the correlation between adhesion molecules levels and mRS score; c′ is the regression coefficient of the correlation between adhesion molecules levels and mRS score, using adhesion molecules levels sICH as independent variables. Abbreviations: mRS, modified Rankin scale; sICH, symptomatic intracranial hemorrhage; and sVCAM-1, soluble vascular cell adhesion molecule-1.

We then utilized the mediation analysis to evaluate whether sICH is partially responsible for the worse functional outcome in patients with higher levels of adhesion molecules. The results of the mediation analysis are demonstrated in Figure 2. We found that sICH mediates the association of sVCAM-1 (*p*-value < 0.001 for the Sobel test) and sE-selectin (*p-*value = 0.007 for the Sobel test) with an mRS score at 90 days. After adding the sICH as an independent variable, the regression coefficient of sVCAM-1 with an mRS score was changed by 25.0% (from 0.280 to 0.210), and the regression coefficient of sE-selectin with an mRS score was changed by 25.5% (from 0.444 to 0.331).

## Discussion

In this sample of patients with stroke who underwent MT, we found that higher levels of sVCAM-1 and sE-selectin were related to an increasing trend of 90-day functional independence and sICH. Moreover, worse functional outcomes associated with the higher adhesion molecules concentrations could be explained partially by post-MT sICH.

To date, the key adhesion molecules, such as ICAM-1, VCAM-1, and E-selectin, have merited special interest in ischemic injury. Soluble isoforms of these molecules are recognized to be shed from the surfaces of activated endothelial cells and leukocytes after ischemic stroke, which can be quantified in cerebrospinal fluid and peripheral blood (29). Previously, several clinical studies have investigated the relationship between levels of adhesion molecules and clinical outcomes in patients with ischemic stroke but yielded contradictory results (12–15). The Chongqing Stroke Study including 238 patients with ischemic stroke indicated that higher levels of sICAM-1, but not of sVCAM-1 and sE-selectin, were associated with early neurological deterioration (12). Furthermore, Rallidis et al. found that increased soluble intercellular adhesion molecule-1 levels are associated with the poor short-term prognosis in middle-aged patients with acute ischemic stroke (15). However, other reports confirmed the value of sE-selectin and sVCAM-1, but not sICAM-1, in predicting the 3-month outcome after cerebrovascular diseases (14). This discrepancy is probably due to the difference in study design, time of assessing adhesion molecules, and definition of outcome. Recently, the BACTRAC program showed that intracranial VCAM1 at the time of endovascular treatment could predict the ischemic stroke severity (16). Our data further demonstrated that patients after MT with higher levels of sVCAM-1 and sE-selectin, seem to have an increasing trend of sICH and worse outcomes. These findings had important clinical implications for early prevention and intervention in patients following reperfusion treatment.

The mechanisms by which adhesion molecules affect clinical outcomes and post-MT complications remain elusive, but several potential pathophysiological pathways have been explored. The dysfunction of the blood-brain barrier may have played a pivotal and initiating role in linking these conditions. ICAM-1, VCAM-1, and E-selectin are prominently expressed on cytokine-activated endothelium and function as cell adhesion molecules (10, 11). Increasing expression of adhesion molecules may mediate the neutrophil recruitment and induce the release of proinflammatory cytokines, which in turn promote the leukocyte-endothelium interaction and damage the endothelial function (10, 30). It is possible that endothelial dysfunction and neurovascular unit injury can contribute to intracerebral hemorrhage and parenchymal damage (18, 31). Other possible pathways include activating microglial cells and leukocyte extravasation, inducing oxidative stress, and promoting a local procoagulant state (18, 32, 33). Further studies are needed to elucidate the underlying biological mechanism.

Despite the accumulating evidence from animal studies that have confirmed the adverse impact of adhesion molecules on ischemic stroke, several large recent trials of treatment aimed at inhibition of neutrophils and anti-adhesion therapy, have been unsuccessful (34, 35). In particular, injection of anti-ICAM-1 is not an effective treatment for ischemic stroke, indeed, may significantly worsen the stroke outcome (34). However, most of the patients in these clinical trials did not receive endovascular treatment, which means that the administration of the drug could not reach the ischemic tissue and perform its pharmacological effects. Taken together our findings, whether the anti-adhesion therapy of VCAM-1 and E-selectin in ischemic stroke after MT is a possible future area of inquiry.

The proportion of patients with rescue therapy in this study, including balloon angioplasty, permanent implantation of a stent, intraarterial thrombolysis, or intraarterial tirofiban infusion, was relatively higher (30.6 vs. 25.7% in the NASA Registry) (36). Due to the fact that more prevalent intracranial atherosclerosis is recognized in Asians, the probability of rescue therapy during EVT may be increased (37).

Our study had several limitations that should be addressed. First, this study enrolled a relatively small sample of Chinese patients, and therefore it was impossible to evaluate whether the results generalize to patients of other ethnicities. Second, several outcome-related variables were not included for mediation analysis, such as infarct volume and malignant cerebral edema. Third, the measurement of adhesion molecules was conducted only once within 24 h after admission. There is a possibility that EVT treatment may have changed the serum levels of these biomarkers. It might be more meaningful to assess the dynamic fluctuation of adhesion molecules after stroke. Finally, it is likely that another adhesion molecule (such as, P-selectin) was related to cardiovascular diseases. However, our study only measured three typical adhesion molecules, such as sICAM-1, sVCAM-1, and sE-selectin, limiting a comprehensive assessment of the associations of adhesion molecules with clinical outcomes in patients after MT. Therefore, our findings should be interpreted with caution.

In summary, adhesion molecules, as measured by sVCAM-1 and sE-selectin, seem to be associated with an increased trend of worse outcomes in patients with ischemic stroke undergoing MT. The association could be partially mediated by the presence of sICH. Further mechanistic research and cohort studies with larger sample sizes are needed to determine the elusive mechanism and confirm these preliminary results.

## Data availability statement

The original contributions presented in the study are included in the article/Supplementary material, further inquiries can be directed to the corresponding authors.

## Ethics statement

The studies involving human participants were reviewed and approved by Jinling Hospital and Nanjing First Hospital. The patients/participants provided their written informed consent to participate in this study.

## Author contributions

JZ and XZ designed the study. XZ, FZ, and WW interpreted data and wrote the manuscript. YE, SC, HC, and HL prepared the tables and figures. HL and TJ did the statistical analyses. YZ and HS screened and extracted the data. JZ and HS supervised the study. All authors have made an intellectual contribution to the manuscript and approved the submission.

## Funding

This work was supported by the National Science and Technology Innovation 2030-Major program of Brain Science and Brain-Inspired Intelligence Research (2021ZD0201807) and the Medical Innovation Team of Jiangsu Province (CXTDA2017030).

## Conflict of interest

The authors declare that the research was conducted in the absence of any commercial or financial relationships that could be construed as a potential conflict of interest.

## Publisher's note

All claims expressed in this article are solely those of the authors and do not necessarily represent those of their affiliated organizations, or those of the publisher, the editors and the reviewers. Any product that may be evaluated in this article, or claim that may be made by its manufacturer, is not guaranteed or endorsed by the publisher.
