# Supplementary material for: Levels of adhesion molecules and clinical outcomes in patients with ischemic stroke after mechanical thrombectomy
Source: Front Neurol. 2022 Sep 29;13:1024162. doi: 10.3389/fneur.2022.1024162 (PMC9556902; doi:10.3389/fneur.2022.1024162)
Supplement: Supplementary file 1 [file Data_Sheet_1.PDF]

Supplementary materials

Table S1. Comparison of baseline data according to patients with and without sICH.

Variables	With sICH, n = 46	Without sICH, n = 264	*P* value
Demographic characteristics			
Age, years	67.0 ± 13.6	68.8 ± 12.2	0.373
Male, n (%)	30 (65.2)	168 (63.6)	0.837
Vascular risk factors, n (%)			
Hypertension	32 (69.6)	184 (69.7)	0.986
Diabetes mellitus	7 (15.2)	65 (24.6)	0.163
Hyperlipidemia	4 (8.7)	26 (9.8)	0.807
Atrial fibrillation	22 (47.8)	118 (44.7)	0.694
Current smoker	16 (34.8)	103 (39.0)	0.586
Coronary heart disease	7 (15.2)	37 (14.0)	0.829
Clinical data			
Systolic blood pressure, mmHg	139.9 ± 21.8	136.4 ± 23.7	0.341
Diastolic blood pressure, mmHg	83.9 ± 14.8	81.7 ± 14.1	0.330
Time from puncture to recanalization, min	384.0 (300.0, 480.0)	357.5 (250.0, 571.0)	0.673
Baseline NIHSS, score	15.5 (12.0, 20.0)	13.0 (10.0, 16.0)	0.003
Baseline ASPECTS, score	8.0 (7.0, 9.0)	9.0 (8.0, 9.0)	<0.001
Stroke etiology, n (%)			0.608
Atherosclerotic	20 (43.5)	128 (48.5)	
Cardioembolic	20 (43.5)	113 (42.8)	
Others	6 (13.0)	23 (8.7)	
Prior intravenous thrombolysis, n (%)	22 (47.8)	114 (43.2)	0.558
Poor collateral status, n (%)	29 (63.0)	126 (47.7)	0.045
Successful reperfusion, n (%)	36 (78.3)	233 (88.3)	0.065
Procedural models, n (%)			0.314
Pure thrombectomy	29 (63.0)	186 (70.5)	
Need for rescue therapy*	17 (37.0)	78 (29.5)	
Passes with retriever	2.0 (1.0, 4.0)	1.0 (1.0, 2.0)	<0.001
Vascular occlusion site, n (%)			0.669
Internal carotid artery	17 (37.0)	89 (33.7)	
Middle cerebral artery	29 (63.0)	175 (66.3)	
Laboratory data			
Baseline blood glucose, mmol/L	8.7 ± 3.4	7.3 ± 2.1	0.017
Hs-CRP, mg/L	9.0 (3.5, 30.5)	10.6 (3.4, 23.0)	0.842
sICAM-1, pg/mL	1.6 (0.8, 2.1)	1.5 (0.8, 12.2)	0.974
sVCAM-1, pg/mL	6.8 (5.3, 9.0)	5.9 (4.4, 7.6)	0.073

sE-Selectin, pg/mL	8.1 (15.2, 11.8)	7.4 (4.5, 9.1)	0.025

Abbreviations: ASPECTS, the Alberta Stroke Program Early Computed Tomography Score; Hs-CRP, hypersensitive C-reactive protein; NIHSS, National Institute of Health Stroke Scale; sE-Selectin, soluble E-selectin; sICAM-1, soluble intercellular adhesion molecule-1; sICH, symptomatic intracranial hemorrhage; sVCAM-1, soluble vascular cell adhesion molecule-1.

*Rescue therapy includes balloon angioplasty, permanent implantation of a stent, intraarterial thrombolysis, or intraarterial tirofiban infusion.

Table S2. Multivariate regression analysis for the associations of adhesion molecule markers with sICH.

	Crude model		Adjusted model	
	OR (95% CI)	*P* value	OR (95% CI)	*P* value
sICAM-1, Per 1-SD increase	0.950 (0.656−1.250)	0.545	1.083 (0.740−1.584)	0.688
sICAM-1 (quartiles)				
First quartile	Ref		Ref	
Second quartile	1.110 (0.438−2.763)	0.839	1.897 (0.600−5.917)	0.276
Third quartile	1.757 (0.742−4.165)	0.200	2.585 (0.661−9.070)	0.126
Fourth quartile	0.874 (0.334−2.285)	0.783	1.672 (0.490−5.707)	0.412
sVCAM-1, Per 1-SD increase	1.239 (0.940−1.634)	0.129	1.202 (0.846−1.790)	0.305
sVCAM-1 (quartiles)				
First quartile	Ref		Ref	
Second quartile	1.054 (0.551−4.109)	0.426	1.943 (0.563−6.705)	0.293
Third quartile	1.767 (0.655−4.768)	0.261	1.921 (0.572−6.488)	0.290
Fourth quartile	2.470 (0.952−6.406)	0.063	2.766 (0.869−8.803)	0.085
sE-Selectin, Per 1-SD increase	1.517 (1.350−2.027)	0.005	1.405 (1.016−1.944)	0.040
sE-Selectin (quartiles)				
First quartile	Ref		Ref	
Second quartile	1.615 (0.621−4.203)	0.326	1.595 (0.549−4.635)	0.391
Third quartile	0.739 (0.244−2.241)	0.594	0.605 (0.184−1.990)	0.408
Fourth quartile	3.017 (1.238−7.535)	0.015	2.422 (0.822−6.648)	0.086

Abbreviations: CI, confidence interval; OR, odd ratio; SD, standard deviation; sE-Selectin, soluble E-selectin; sICAM-1, soluble intercellular adhesion molecule-1; sICH, symptomatic intracranial hemorrhage; sVCAM-1, soluble vascular cell adhesion molecule-1.

The adjusting model was controlled for demographic characteristics and variables with a *P* value < 0.1 in the univariate analysis including baseline NIHSS score, pre-treatment ASPECTS, poor collateral status, successful reperfusion, sICH, passes with retriever and baseline blood glucose.
